# The Association Between Breast Density and Gut Microbiota Composition at 2 Years Post-Menarche: A Cross-Sectional Study of Adolescents in Santiago, Chile

**DOI:** 10.3389/fcimb.2021.794610

**Published:** 2021-12-17

**Authors:** Lara S. Yoon, Jonathan P. Jacobs, Jessica Hoehner, Ana Pereira, Juan Cristóbal Gana, Camila Corvalán, Karin B. Michels

**Affiliations:** ^1^ Department of Epidemiology, Fielding School of Public Health, University of California, Los Angeles, CA, United States; ^2^ Vatche and Tamar Manoukian Division of Digestive Diseases, Department of Medicine, David Geffen School of Medicine, University of California, Los Angeles, CA, United States; ^3^ Division of Gastroenterology, Hepatology and Parenteral Nutrition, Veterans Administration Greater Los Angeles Healthcare System, Los Angeles, CA, United States; ^4^ Leidos, Inc., Atlanta, GA, United States; ^5^ Institute of Nutrition and Food Technology, University of Chile, Santiago, Chile; ^6^ Department of Pediatric Gastroenterology and Nutrition, Division of Pediatrics, School of Medicine, Pontificia Universidad Católica de Chile, Santiago, Chile; ^7^ Institute for Prevention and Cancer Epidemiology, Faculty of Medicine and Medical Center, University of Freiburg, Freiburg, Germany

**Keywords:** adolescence, human, epidemiology, breast density, breast cancer, gut microbiota, 16s rRNA

## Abstract

The gut microbiome has been linked to breast cancer *via* immune, inflammatory, and hormonal mechanisms. We examined the relation between adolescent breast density and gut microbial composition and function in a cohort of Chilean girls. This cross-sectional study included 218 female participants in the Growth and Obesity Cohort Study who were 2 years post-menarche. We measured absolute breast fibroglandular volume (aFGV) and derived percent FGV (%FGV) using dual energy X-ray absorptiometry. All participants provided a fecal sample. The gut microbiome was characterized using 16S ribosomal RNA sequencing of the V3-V4 hypervariable region. We examined alpha diversity and beta diversity across terciles of %FGV and aFGV. We used MaAsLin2 for multivariable general linear modeling to assess differential taxa and predicted metabolic pathway abundance (MetaCyc) between %FGV and aFGV terciles. All models were adjusted for potential confounding variables and corrected for multiple comparisons. The mean %FGV and aFGV was 49.5% and 217.0 cm^3^, respectively, among study participants. Similar median alpha diversity levels were found across %FGV and aFGV terciles when measured by the Shannon diversity index (%FGV T1: 4.0, T2: 3.9, T3: 4.1; aFGV T1: 4.0, T2: 4.0, T3: 4.1). %FGV was associated with differences in beta diversity (*R^2 =^* 0.012, *p*=0.02). No genera were differentially abundant when comparing %FGV nor aFGV terciles after adjusting for potential confounders (q > 0.56 for all genera). We found no associations between predicted MetaCyc pathway abundance and %FGV and aFGV. Overall, breast density measured at 2 years post-menarche was not associated with composition and predicted function of the gut microbiome among adolescent Chilean girls.

## Introduction

Breast cancer is the most common cancer among women worldwide, however, one third of affected women have no known or suspected risk factors. Greater breast density is strongly associated with increased risk of breast cancer among adult women (Boyd et al., 1998). Breast density, measured as the relative proportion of fibroglandular tissue to fatty tissue in the breast, is inversely related to age, parity, and later menopause and also associated with childhood and adolescent body fatness (Martin and Boyd, 2008; Harris et al., 2011). Initial peak breast density is hypothesized to be established during adolescence (Boyd et al., 2009; Ghadge et al., 2020), and breast tissue may be particularly vulnerable to exposures during puberty, a period of rapid breast development (Ruder et al., 2008). Adolescent breast density has been associated with pubertal maturation and body fatness (Novotny et al., 2011). However, few studies have characterized other important associates of breast development and composition during this vulnerable period.

A novel mechanistic pathway that may contribute to development of the breast is the gut microbiome. Collectively the trillions of microbes living in the human intestinal tract, the gut microbiome plays important roles in numerous biological processes including immune regulation, dietary metabolism, epithelial barrier function, and hormone regulation (Ley et al., 2006; Turnbaugh et al., 2006; Belkaid and Hand, 2014; Martin et al., 2019). Experimental and epidemiologic evidence suggests that the gut microbiome may be associated with different diseases such as metabolic disorders and cancers, including breast cancer, through direct and indirect mechanisms (Selber-Hnatiw et al., 2017; Fernández et al., 2018; Chen et al., 2019; Eslami-S et al., 2020; Laborda-Illanes et al., 2020; Sepich-Poore et al., 2021). For instance, perturbations in microbiota composition, or dysbiosis, may lead to systemic inflammation resulting in an increased vulnerability to pathogens (Zitvogel et al., 2015). Carcinogenesis may follow from dysbiosis-induced permeability of the intestinal epithelium and resulting extracellular vesicle circulation throughout the body in biofluids (Ricci et al., 2020). Changes in the composition of gut microbiome, and specifically in microbes involved in estrogen metabolism, may influence breast cancer development through an increase in circulating estrogen levels (Goedert et al., 2015). Evidence for an association between circulating estrogens and adult mammographic breast density is mixed (Boyd et al., 2002; Fuhrman et al., 2012; Gierach et al., 2015; Jones et al., 2019). However, we have shown that levels of prepubertal estrogen, measured with an ultra-sensitive method, are associated with earlier thelarche, which is in turn related to breast density at the end of puberty (Pereira et al., 2015). Estrogen levels are directly associated with breast development; therefore, the gut microbiome may play a mechanistic role in the development of breast density during puberty and adolescence (Pereira et al., 2015; Baker et al., 2017).

It is unknown whether the gut microbiome is associated with breast density in adolescence. The current study examined the association between breast density at two years post-menarche and the gut microbiome in a cohort of adolescent Chilean girls. We hypothesized that the microbial composition and function of the gut would differ across densities of the breast.

## Materials and Methods

### Study Design and Population

We conducted a cross-sectional analysis of a subset of female participants in the Growth and Obesity Cohort Study (GOCS). The original GOCS, which began in 2006, has been described previously (Corvalán et al., 2009). In brief, 1,196 children aged 2.5 to 4 years from low- and middle- income families in the South East region of the Santiago metropolitan area in Chile and enrolled in preschool at the National Board of Preschool Council Program (Junta Nacional de Jardines Infantiles) were enrolled in the study; approximately half (601) were girls. Participants in the study visit the Institute of Nutrition and Food Technology (INTA) Health Clinic at the Universidad de Chile in Santiago, Chile at least once per year for anthropometric assessments, pubertal (Tanner) evaluation, collection of biospecimens, and to complete 24-hour dietary recall interviews. A limited set of behavioral and demographic information was also collected *via* questionnaire. Breast composition was measured when the participants were two years post-menarche (2PM). The current study included a subset of 218 girls randomly selected to provide a fecal sample and who had a breast composition measurement at 2PM. The 2PM breast assessment and fecal sample collection occurred between 2018 and 2019. The study protocol and written consent forms were approved by the University of Chile Ethics Committee at INTA.

### Breast Composition Measurement

Breast composition was measured at the clinic visit corresponding to a timepoint of 2PM for each girl using the dual energy X-ray absorptiometry (DXA) breast scanning protocol developed by Shepherd et al. at the University of California, San Francisco (Shepherd et al., 2006). The Prodigy DXA system software (version 13.6, series 200674; GE Healthcare) was used to scan each breast for quantification of adipose fat and fibroglandular (FG) tissue. Stable calibration of the system was continuously performed using a quality control breast phantom. Absolute fibroglandular volume (FGV; cm3) and total breast volume for each breast were derived from a two-compartment model of adipose fat and fibroglandular tissue. The percentage of FGV tissue in the breast was derived by dividing the absolute FGV by total breast volume and multiplying by 100. Percent FGV and absolute FGV for the left and right breast were averaged to obtain two single measures of breast density: percent FGV (%FGV) and absolute FGV (aFGV). DXA is frequently used in studies of bone density in children; exposure to ionizing radiation from the DXA protocol is low (Crabtree et al., 2014). The DXA approach for breast composition assessment has high validity and precision among adolescent girls (Shepherd et al., 2008). Both breast density outcomes were categorized into terciles (‘T1’, ‘T2’, ‘T3’) based on the distribution of the sample for statistical analyses.

### Fecal Collection

Fecal samples were collected after annual visits to the INTA health clinic occurring when the girls were between 13 and 15 years old. During the visit, girls were provided with materials (sealed plastic bag, stool catcher, a plastic, sterile container with a spoon to manipulate the sample and procedure gloves) and instructions for at-home fecal collection. Briefly, after the stool deposit, girls were asked to collect a part of the stool approximately the size of a walnut and place it in the plastic sterile container and label with the date and time of sample collection. Within 15 minutes of collection, the samples were sealed and then stored temporarily in the participant’s freezer, at which time study personnel were contacted to schedule the sample pick up. Samples were retrieved from the participant’s home no later than three days after initial collection, transported on ice to INTA, labeled with a de-identified key, and stored in a -80°C freezer prior to shipment. All stool samples were shipped on dry ice to the National Exposure Assessment Laboratory at Emory University.

### Fecal Processing and 16s rRNA Sequencing

Fecal processing of 279 samples (266 unique samples and 13 duplicates) was performed at the National Exposure Assessment Laboratory at Emory University, a Children’s Health Exposure Analysis Resource Laboratory. DNA was extracted from fecal samples using the Qiagen DNeasy PowerSoil Kit (Qiagen; 12888) with the TissueLyser at maximum speed (30 beats per second) for 10 minutes. Composition of the gut microbiome was determined through sequencing and amplification of the V3-V4 hypervariable region of the 16S rRNA gene (PCR primer pair: 341F 5’-GTGCCAGCMGCCGCGGTAA-3’; 805R 5’-GACTACHVGGGTWTCTAAT-3’). Libraries were made from 12.5 ng of DNA following a standard 16S Metagenomic Library Preparation Workflow from Illumina, Inc. Libraries were pooled in equal amounts based on fluorescence quantification, resulting in a 630 bp amplicon. Final library pools were quantitated *via* qPCR (Kapa Biosystems; KK4824). The pooled library was sequenced on an Illumina MiSeq using MiSeq v3 600 cycle chemistry (Illumina MS-102-3003) at a loading density of 6-8 pM with 20% PhiX, generating roughly 20M, 300 bp paired-end reads. In addition to the 279 experimental samples, traditional negative, no template control (NTC) negative, positive, and ZymoBIOMICS mock microbial community controls were included in the assays (Pollock et al., 2018). The Emory Integrated Genomics Core performed the assays.

After sequencing, demultiplexed raw amplicon sequences were processed using QIIME2 (Quantitative Insights Into Microbial Ecology) version 2019.4 (Bolyen et al., 2019). Denoising and dereplication, including chimera removal and trimming of reads based on Phred quality scores, were performed using the Divisive Amplicon Denoising Algorithm 2 (DADA2) module (Callahan et al., 2016a). The majority of Phred scores were greater than 25. To improve sensitivity of the algorithm, we trimmed the first 30 base pairs and truncated the reads at position 290, where the mean read quality distribution dropped for the overall dataset. Amplicon sequence variants (ASVs) were inferred using DADA2 to increase resolution and allow for intrinsic biologic meaning (Callahan et al., 2017). Taxonomy was assigned using a naive Bayes classifier on the SILVA database (SILVA 132 release) (Quast et al., 2013). We excluded samples from girls that did not have breast assessments at 2PM (n=48). The end product yielded a total of 6,270 unique ASVs from 218 samples. Standard preprocessing (filtering, subsetting, agglomeration) with the R package ‘phyloseq’ was used to exclude undefined (NA) or ambiguous (uncharacterized) phyla, phyla below a prevalence of 1, and ASVs below a prevalence threshold of 2% of total samples (McMurdie and Holmes, 2013; Callahan et al., 2016b). The final ASV feature table comprised 18,628,903 total reads (mean per sample = 87,872, range 28,118 to 278,065) and a total of 1,600 unique ASVs across 218 samples.

### Covariates

Demographic, anthropometric, and nutritional data were collected by trained dietitians during the annual study visit at the health clinic. Age- and sex- adjusted body mass index (BMI; kg/m^2^) Z-scores were calculated using the World Health Organization growth reference data and categorized into Norma/Underweight (Z-score ≤ 1), Overweight (1 < Z-score ≤ 2), and Obese (2 < Z-score). Body fat percentage was estimated using Tanita-BC-418 MA bioelectrical impedance measurements (Tanita-Corporation, Tokyo, Japan) and categorized into Healthy, Overweight, and Obese based on Tanita children’s age- and sex-specific body fat reference curves (McCarthy et al., 2006; Cediel et al., 2016). Age at menarche was determined *via* phone interviews completed by study dietitians every three months during puberty and dichotomized (≤ 12 years, > 12 years). Mothers of the participants were present at the clinic visits and were asked to complete short questionnaires with information on their highest level of education (secondary education or less, post-secondary education), birth mode of the participant (cesarean, vaginal), and months of exclusive breast feeding (<3 months, 3-6 months, >6 months). Dietary 24-hour recalls were collected longitudinally beginning in April 2014 by trained dietitians using the USDA multiple-pass method. Food and nutrient information were obtained using a harmonization process that mapped Chilean foods to the USDA Food and Nutrient Database for Dietary Studies. Daily intake of five major food groups (vegetables [g], fruit [g], red or processed meat [g], yogurt [g], and whole grains [g]) and total energy intake (kCal) were averaged over the data collection period and up to the date of the 2PM breast composition assessment to reduce random-measurement error and to obtain a more accurate assessment of long-term diet (Willett, 2012). We also collected data on ethnicity (Mapuche [native Chilean indigenous] and non-Mapuche, according to last name), average daily hours of television during the week (≤1 hour, 1-3 hours, >3 hours) as a proxy of physical activity, and antibiotic use in the 6 months prior to fecal sample (yes, no). Missing covariate data were imputed using last observation carried forward for body fat percentage and dietary variables. We assumed the remaining covariates with missing values, including maternal education, ethnicity, breast feeding, birth mode, antibiotic use, and television hours were missing completely at random. We used mean imputation (breast feeding) or median imputation (maternal education, ethnicity, birth mode, antibiotic use, television hours) in the R package ‘mice’ (van Buuren and Groothuis-Oudshoorn, 2011). The variable with the highest proportion of missing values was television hours (n=22, 10.1%); all other covariates were missing at a proportion less than 3%.

### Statistical Analysis

Relative abundance at the phylum level was plotted for all samples and across %FGV and aFGV terciles. We estimated alpha diversity as the observed richness (the number of species per sample) and the Shannon index (a measure of richness and evenness) on unfiltered data (Shannon, 1948; Chao, 1987). Rarefaction without replacement was used to standardize library sizes and account for uneven sampling depth prior to estimating alpha and beta diversity metrics and has been suggested to be suitable for microbiome data (McKnight et al., 2019; Estaki et al., 2020). Overall differences in observed richness and the Shannon index by %FGV and aFGV terciles were tested using the Kruskal-Wallis (KW) followed by *post-hoc* pairwise testing with the Wilcoxon Rank Sum in cases where data were compatible with evidence to reject the KW null hypothesis. To evaluate whether our results were sensitive to body composition, we performed analysis of covariance (ANCOVA) and adjusted for body fat percentage. Beta diversity was visualized using principal coordinate analysis (PCoA) plots with Bray-Curtis dissimilarity. Single and multivariate permutational analysis of variance (PERMANOVA) were used to test for overall differences in microbial composition between %FGV and aFGV terciles and produced marginal effects using the adonis2 function in the ‘vegan’ package (McArdle and Anderson, 2001; Oksanen et al., 2007). Homogeneity of variance was used to test whether differences in community structure were due to dissimilar dispersions. Supplemental PCoA visualization and PERMANOVA tests were done using weighted UniFrac, which differs from Bray-Curtis in that it incorporates phylogenetic relationships (Lozupone et al., 2007). Dietary intake of whole grains, fruit, vegetables, and yogurt are important predictors of gut microbial composition and may influence breast density. Therefore, we performed supplementary PERMANOVA analyses with added terms for four food-specific intake groups, including average whole grain intake (g/day), average fruit intake (g/day), average vegetable intake (g/day), and average yogurt intake (g/day). Microbial diversity metrics were estimated using the R packages ‘phyloseq’ and ‘vegan’ (Oksanen et al., 2007; McMurdie and Holmes, 2013).

ASVs from the filtered final feature table were agglomerated to the genus level prior to associating microbial community features with breast density outcomes. A total of 223 genera remained after agglomeration of the 1,600 ASVs. We used the R package ‘MaAsLin2’ (Microbiome Multivariable Associations with Linear Models), which relies on a modified generalized linear model for compositional data, to identify differentially abundant microbe genera across %FGV and aFGV terciles (Mallick et al., 2021). All models specified minimum abundance of 0.01% in 10% of samples, total sum scaling normalization, and arcsine square root-transformation (Mallick et al., 2021). The models were adjusted for a set of potential confounders including age, body fat percentage, antibiotic use, maternal education, total calories, hours of TV watching, ethnicity, birth mode, and breast feeding. Where appropriate, the Benjamini and Hochberg (BH) correction method was used to control the false discovery rate (FDR) and produced q-values (Benjamini and Hochberg, 1995). Supplementary MaAsLin2 analyses were also performed with adjustment for the four food-specific dietary intake groups.

Functional metabolic pathways of microbial communities were predicted from 16S rRNA marker sequencing data using PICRUSt2 implemented as a QIIME2 plugin (Douglas et al., 2020, 2). Default parameters were used in the pipeline, including SATe-enabled phylogenetic placement, hidden-state prediction, and a distance cut-off of 2. PCoA ordination with Bray-Curtis dissimilarity was used to visualize predicted abundance of metabolic pathways present in the MetaCyc Metabolic Pathway Database. Differences in MetaCyc pathway abundance between %FGV and aFGV terciles were testing using multivariable PERMANOVA. Multivariable MaAsLin2 was used to evaluate differential abundance of 458 predicted MetaCyc pathways with default parameters and BH correction for multiple comparisons. As in prior analyses, we additionally adjusted for the four food-specific dietary intake groups in supplementary analyses.

## Results

### Study Sample Characteristics

The association between breast density and gut microbial composition and function was assessed among 218 GOCS participants. The mean age of the participants was 14 years at the time of breast assessment and 12 years at reported menarche (**Table 1**). The participants were primarily non-indigenous (Non-Mapuche, 83.0%) and 78.4% of the mothers had a secondary school educational attainment or less. The mean %FGV among the sample was 49.5% (SD=14.5); for tercile 1 (T1), mean %FGV was 34.0%; tercile 2 (T2), 48.4%; and tercile 3 (T3), 65.3%. The mean aFGV among the sample was 217.0 cm3; for T1, mean aFGV was 140.2 cm^3^; T2, 210.8 cm^3^; and T3, 292.7 cm^3^. More than half the cohort was considered overweight or obese. Overall, participants had a mean BMI Z-score of 1.0 and a mean body fat percentage of 32.6%. Additional study population characteristics are reported in **Table 1**.

**Table 1:** Population characteristics of 218 girls participating in the Growth and Obesity Cohort Study.

Characteristic	Distribution
Percent FGV (%), mean (SD)	49.5 (14.5)
Tercile 1, median (range)	34.0 (19.1, 41.4)
Tercile 2, median (range)	48.8 (41.5, 56.3)
Tercile 3, median (range)	65.3 (56.6, 98.0)
Absolute FGV (cm3), mean (SD)	217.0 (78.1)
Tercile 1, median (range)	140.2 (74.2, 178.0)
Tercile 2, median (range)	210.8 (178.1, 249.2)
Tercile 3, median (range)	292.7 (250.1, 546.4)
Total breast volume (cm^3^), mean (SD)	472.0 (236.4)
Age (years), mean (SD)	14.0 (0.9)
Age at menarche (years), mean (SD)	12.0 (0.8)
BMI Z-score, mean (SD)	1.0 (1.0)
Body fat percentage (%), mean (SD)	32.6 (5.8)
Energy intake per day (kCal), mean (SD)	1709.4 (376.5
Ethnicity, n (%)	
Non-Mapuche	181 (83.0)
Mapuche	37 (17.0)
Birth mode, n (%)	
Cesarean	54 (24.8)
Vaginal	164 (75.2)
Antibiotic use in prior 6 months, n (%)	
No	198 (90.8)
Yes	20 (9.2)
Maternal education, n (%)	
Secondary education or less	171 (78.4)
Post-secondary education	47 (21.6)
Hours of TV per day, n (%)	
≤1 hour	77 (35.3)
1-3 hours	109 (50.0)
>3 hours	32 (14.7)
Months of exclusive breast feeding, n(%)	
<3 months	64 (29.4)
3-6 months	129 (59.2)
>6 months	25 (11.5)

### Phylum-Level Microbial Composition of the Sample

**Figure 1:**
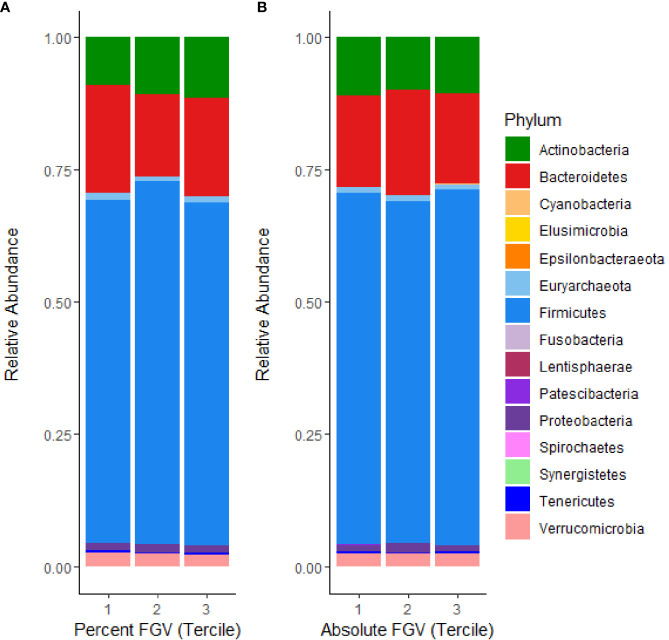
Microbial mean relative abundance at the phylum level stratified by **(A)** %FGV tercile and **(B)** aFGV tercile in fecal microbiota samples from 218 GOCS participants.

The relative composition of the gut microbiome for each %FGV and aFGV tercile is displayed in **Figure 1**. Overall, *Firmicutes* was the most highly represented bacterial phylum and comprised 66% of the sample abundance. Microbes from the *Bacteroidetes* and *Actinobacteria* phyla represented 18.1% and 10.4% of the abundance, respectively. We found minor differences in relative abundance across %FGV terciles for *Bacteroidetes* (T1: 20.4%, T2: 15.4%, T3: 18.5%), *Actinobacteria* (T1: 8.9%, T2: 10.9%, T3: 11.5%) and *Euryarchaeota* (T1: 1.4%, T2: 0.9%, T3: 0.9%) (**Supplementary Figure 1**). There were no differences in relative abundance at the phylum level across aFGV terciles (**Supplementary Figure 2**).

### Global Microbial Diversity Analyses

**Figure 2:**
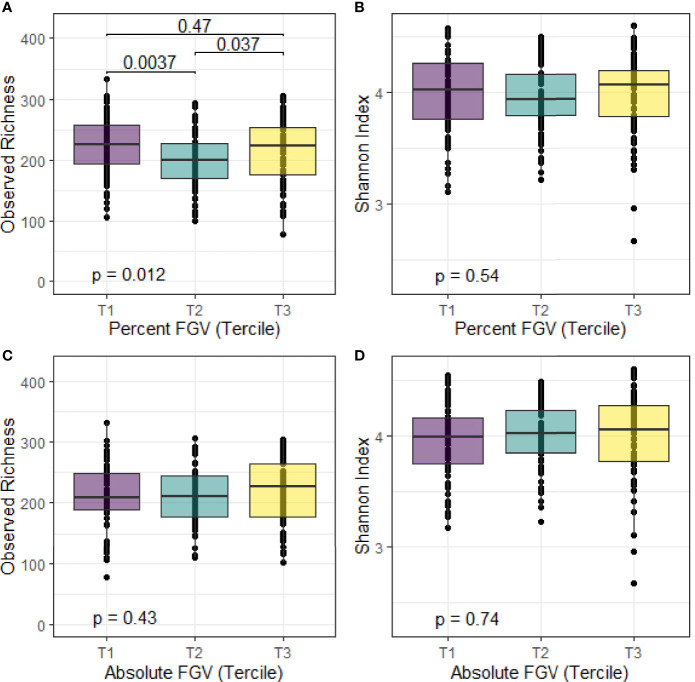
Box plots of alpha diversity metrics for observed richness and Shannon index across terciles of %FGV **(A, B)** and aFGV **(C, D)**. P-values are presented for overall differences in alpha diversity metrics (Kruskal-Wallis) and *post-hoc* pairwise differences. Boxes represent the lower, median, and upper quartile of the data and whiskers are 1.5*interquartile range.

We calculated alpha diversity for each sample using observed richness and the Shannon diversity index. Overall, the mean number of observed species present in the fecal samples was 214 and the mean Shannon index was 4.0 (**Figure 2**). We observed differences in median observed species richness across %FGV terciles (T1: 226; T2: 200, T3: 223). There were no differences in the observed number of species across aFGV terciles nor in the Shannon index for both %FGV and aFGV terciles. Results from ANCOVA did not suggest a difference in alpha diversity across %FGV nor aFGV terciles when adjusting for body fat percentage (data not shown).

**Figure 3:**
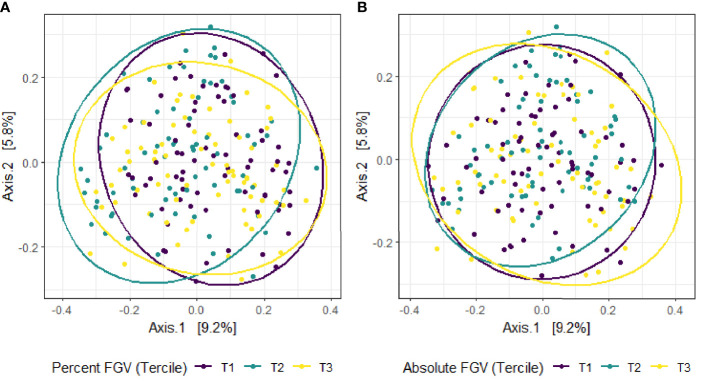
Principal coordinate analysis (PCoA) plot of microbial composition derived from Bray-Curtis dissimilarity among fecal samples provided by 218 girls in GOCS colored by %FGV **(A)** and aFGV **(B)** terciles. Ellipses are 95% confidence regions for each tercile.

**Figure 4:**
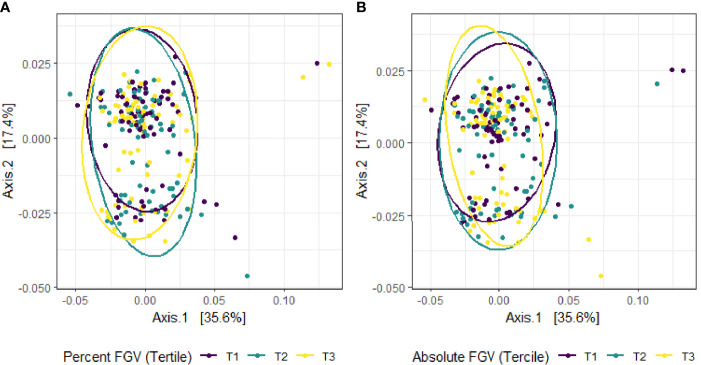
Principal coordinate analysis (PCoA) plot of predicted MetaCyc pathway abundance derived from Bray-Curtis dissimilarity among fecal samples provided by 218 girls in GOCS colored by %FGV **(A)** and aFGV **(B)** terciles. Ellipses are 95% confidence regions for each tercile.

Beta diversity analysis for microbial ASV abundance was performed using Bray-Curtis dissimilarity and visualized by principal coordinate analysis (PCoA) (**Figure 3**). PCoA axes 1 and 2 represented 9.2% and 5.8% of the total variance, respectively. Results from multivariable PERMANOVA analyses suggested that a small but statistically significant portion of the variability in microbial composition was associated with %FGV (*R^2 ^= *0.012, *p*=0.02) (**Table 2**). No significant contribution to variability was associated with aFGV (*R^2 ^= *0.09, *p*=0.53). We also did not note any visual shifts in global predicted microbial pathway abundances (**Figure 4**) nor any significant predictors of Bray-Curtis dissimilarity in PERMANOVA analyses (**Table 3**). In supplementary analyses of beta diversity using weighted UniFrac, rather than Bray-Curtis, we found no significant association with %FGV and aFGV (**Supplementary Table 1**). Supplementary PERMANOVA analyses with additional adjustment for dietary predictors did not substantially modify the variation in microbial nor pathway diversity (**Supplementary Tables 2**, **3**).

**Table 2:** Multivariable PERMANOVA analyses to identify variation (R^2^) in microbial beta diversity (Bray-Curtis dissimilarity) explained by study characteristics.

Characteristic^a^	*R^2^*	p-value	*R^2^*	p-value
Percent FGV	0.012	0.02	–	–
Absolute FGV	–	–	0.009	0.53
Body fat percentage	0.010	0.23	0.011	0.10
Age	0.005	0.24	0.005	0.27
Antibiotic use	0.006	0.15	0.005	0.15
Birth mode	0.004	0.78	0.004	0.81
Breast feeding	0.009	0.56	0.009	0.53
Daily caloric intake	0.013	0.60	0.013	0.59
Maternal education	0.005	0.28	0.005	0.25
Ethnicity	0.007	0.03	0.006	0.05
TV hours	0.003	1.00	0.003	1.00
*Residuals*	0.926		0.929	

^a^Table presents marginal effects for the association of study characteristics to microbial community structure, such that each term was analyzed in a model with all the other variables.

**Table 3:** Multivariable PERMANOVA analyses to identify variation (R^2^) in MetaCyc predicted pathway beta diversity (Bray-Curtis dissimilarity) explained by study characteristics.

Characteristic^a^	*R^2^*	p-value	*R^2^*	p-value
Percent FGV	0.008	0.59	–	–
Absolute FGV	–	–	0.006	0.76
Body fat percentage	0.010	0.31	0.009	0.41
Age	0.004	0.54	0.004	0.57
Antibiotic use	0.006	0.19	0.006	0.18
Birth mode	0.003	0.79	0.003	0.74
Breast feeding	0.009	0.46	0.008	0.57
Daily caloric intake	0.017	0.21	0.018	0.19
Maternal education	0.007	0.20	0.007	0.18
Ethnicity	0.004	0.52	0.003	0.64
TV hours	0.010	0.30	0.010	0.31
*Residuals*	0.921		0.922	

^a^Table presents marginal effects for the association of study characteristics to microbial community structure, such that each term was analyzed in a model with all the other variables.

### Differential Abundance of Microbial Taxa and Predicted Functional Pathways

In multivariable linear modeling with MaAsLin2, no genera were associated with %FGV nor with aFGV after FDR correction (**Supplementary Table 4**). These null associations remained after further adjustment for additional dietary predictors, including whole grain, fruit, vegetable, and yogurt intake (**Supplementary Table 5**). Additionally, there were no major differences in abundance of predicted MetaCyc pathways by %FGV nor aFGV when adjusting for potential confounders (**Supplementary Table 6**) or important dietary predictors (**Supplementary Table 7**).

## Discussion

In this cross-sectional study of Chilean adolescents, we found primarily null associations of microbial composition and predicted function with breast density. Our results suggest minimal differences in alpha diversity: girls in the lowest and highest breast %FGV terciles had slightly higher observed species richness compared to those in tercile 2. However, this pattern was not observed for aFGV and when examining different alpha diversity metrics (Shannon index). We also noted a small but significant contribution of breast %FGV to beta diversity.

Several recent clinical studies have examined the association between the gut microbiome and breast cancer. In a case-control study of 96 post-menopausal women, pre-treatment breast cancer patients had altered microbial composition (beta diversity) and increased abundance of *Clostridiaceae*, *Faecalibacterium*, and *Ruminococcaceae* compared to controls (Goedert et al., 2015). A cross-sectional study of incident pre- and post-menopausal breast cancer patients reported a less diverse microbiome and differential abundance of *Firmicutes* in women with human epidermal growth factor receptor 2 (HER2) positive breast cancer compared to HER2 negative breast cancer (Wu et al., 2020). Other studies also support associations of specific microbial taxa (e.g., *Bacteroidetes*, *Blautia* spp.) with breast cancer staging and clinical characteristics, including body size (Bard et al., 2015; Luu et al., 2017). Much less information is available from epidemiologic studies on the possible association between the gut microbiome and breast density. A study of healthy menopausal women in the United States found no association between mammographic breast density and gut microbial beta diversity and *Firmicutes* to *Bacteroidetes* (F/B) ratio, and suggestive but non-significant differences in alpha and beta diversity (Yaghjyan et al., 2021). These results are comparable to those from a study of cancer-free postmenopausal women, which found that alpha diversity and relative abundance did not differ in women with high versus low mammographic density (Jones et al., 2019). To our knowledge, our study is the first that examines the association between the gut microbiome and breast density in adolescents. Our study differs in several ways from the prior studies of the breast density and gut microbiome relation, notably in study population and breast density assessment method. In contrast to the studies from Yyghjyan and Jones, we found small but significant differences in beta diversity across terciles of breast density. However, we also found similar null associations for alpha diversity and relative abundance with respect to breast density alone. Null findings specifically in studies of the gut microbiome-breast density association might reflect the complex interaction between body composition (e.g., body fatness), the gut microbiome, and breast density. Obesity is strongly inversely associated with breast density and with diversity of the gut microbiome (Ley et al., 2006; Turnbaugh et al., 2006; Dorgan et al., 2012). It is plausible that an association is mediated by body composition, such that any effect is cancelled out when controlling for body fat percentage (Boyd et al., 2011). However, we did not note any breast density and microbial composition associations in unadjusted analyses. It is also possible that studies of the microbiome-breast cancer association are reflective of alterations to the composition of the gut following the disease state, rather than the hypothesized effects of the gut microbiome in contributing to breast cancer pathogenesis through early alteration of estrogen metabolism.

Our study has several limitations. The cross-sectional design does not allow for a temporal specification of the gut microbiome – breast density association. However, the human gut microbiome is thought to be relatively stable after infancy and early childhood (Lozupone et al., 2012). We cannot fully preclude that other perturbations of the microbiome, such as major dietary changes, occurred in the time just prior to sample collection. However, we collected information on antibiotic usage and clinical diagnoses of disease in the six months prior to stool collection, and less than 2% of the sample reported any probiotic consumption. We did not have comprehensive information on nutritional supplements; however, use of vitamins or nutritional supplements is uncommon in Chile, particularly among girls. We had limited information on physical activity, which is associated with gut microbial composition and many anthropometric characteristics (e.g., body fatness) associated with breast density. We also lack direct information on functional microbiome data which may show relations with breast density that were not apparent when examining composition alone. However, we were able to approximate functional potential of the community using PICRUSt2.

Our study also has several strengths, including a large sample of geographically, socioeconomically, and behaviorally similar girls. We were also able to comprehensively assess the gut microbial community using fecal 16s rRNA gene sequencing. The GOCS study has collected longitudinal data on lifestyle, socioeconomic, and anthropometric factors, allowing for specific control of potential confounders. MaAsLin2, a novel general linear modeling approach, allows for preserved statistical power with multiple covariates and control for false-discovery rate.

In conclusion, we found no important association between the gut microbiome and breast density at 2 years post-menarche in female adolescents.

## Data Availability Statement

The datasets presented in this study can be found in online repositories. The names of the repository/repositories and accession number(s) can be found below: https://www.ncbi.nlm.nih.gov/ (SRA, BioProject PRJNA744878), https://www.doi.org/10.36043/1977_490.

## Ethics Statement

The studies involving human participants were reviewed and approved by Universidad de Chile Instituto de Nutrición y Tecnología de los Alimentos and the University of California, Los Angeles. Written informed consent to participate in this study was provided by the participants’ legal guardian/next of kin.

## Author Contributions

LSY designed the study, analyzed the data, interpreted the results, and wrote the manuscript. KBM conceived the study, contributed resources, and interpreted the study results. JPJ and JH analyzed the data and interpreted the study results. AP, JC, and CC collected biospecimen samples and other study data and contributed resources. All authors contributed to the article and approved the submitted version.

## Funding

This research was supported by research grant U01ES026130 from the National Institute of Environmental Health and the National Cancer Institute, National Institutes of Health (to KBM). This research was also supported by the National Institute of Environmental Health Sciences of the National Institutes of Health under Award Number U2CES026560 to Emory University and the Emory Integrated Computational Core (EICC), which is subsidized by the Emory University School of Medicine and is one of the Emory Integrated Core Facilities. Additional support was provided by the National Center for Georgia Clinical & Translational Science Alliance of the National Institutes of Health under Award Number UL1TR002378. LSY was supported by T32 training grant 5T32CA009142 from the National Cancer Institute, National Institutes of Health and the Karen Toffler Charitable Trust. JPJ was supported by VA CDA2 IK2CX001717. The content is solely the responsibility of the authors and does not necessarily reflect the official views of the National Institutes of Health. The article processing charge was funded by the Baden-Wuerttemberg Ministry of Science, Research and Art and the University of Freiburg in the funding programme Open Access Publishing.

## Author Disclaimer

The content is solely the responsibility of the authors and does not necessarily represent the official views of the National Institutes of Health.

## Conflict of Interest

Author JH is employed by Leidos, Inc.

The remaining authors declare that the research was conducted in the absence of any commercial or financial relationships that could be construed as a potential conflict of interest.

## Publisher’s Note

All claims expressed in this article are solely those of the authors and do not necessarily represent those of their affiliated organizations, or those of the publisher, the editors and the reviewers. Any product that may be evaluated in this article, or claim that may be made by its manufacturer, is not guaranteed or endorsed by the publisher.
