# Supplementary material for: The Association Between Breast Density and Gut Microbiota Composition at 2 Years Post-Menarche: A Cross-Sectional Study of Adolescents in Santiago, Chile
Source: Front Cell Infect Microbiol. 2021 Dec 17;11:794610. doi: 10.3389/fcimb.2021.794610 (PMC8718921; doi:10.3389/fcimb.2021.794610)
Supplement: Supplementary file 1 [file Image_1.pdf]

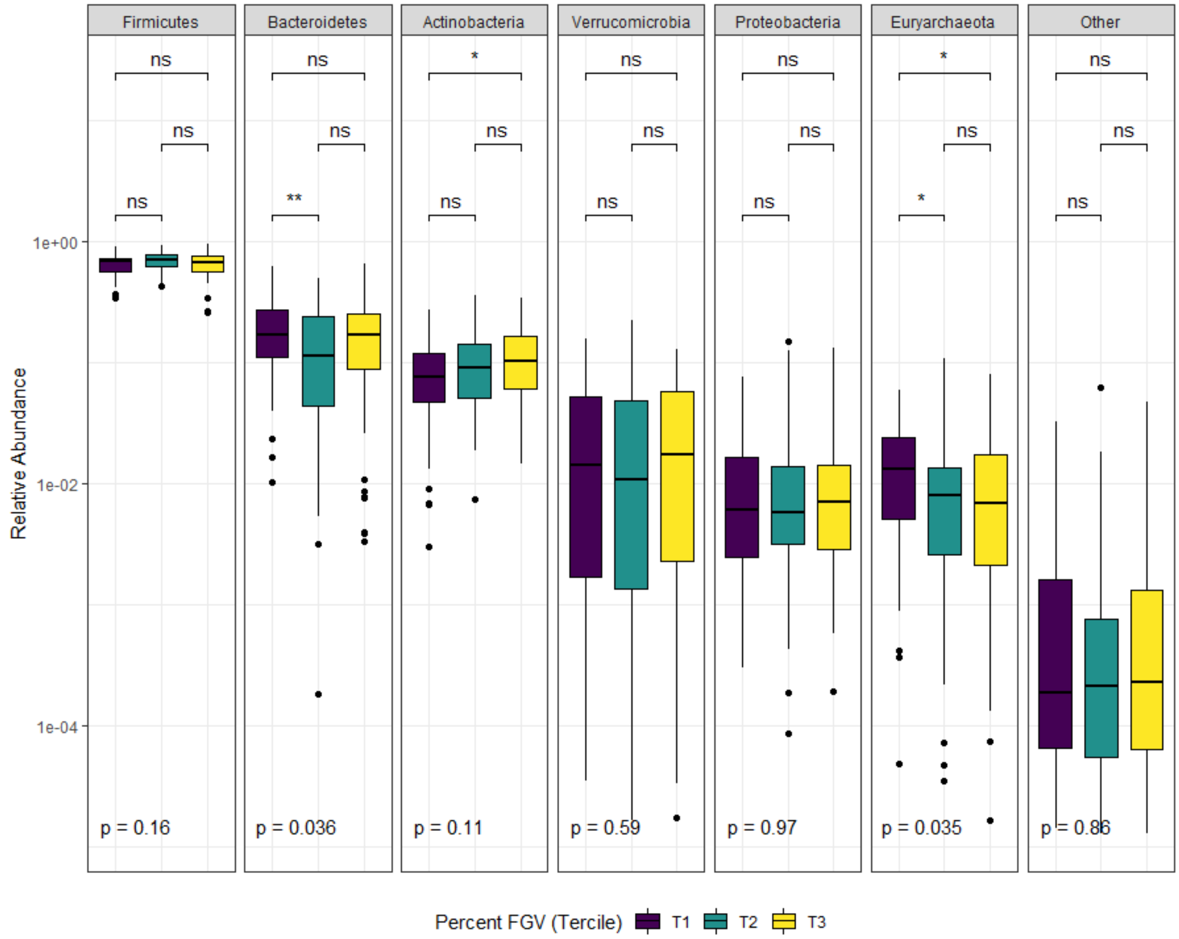

Supplementary Figure 1. Relative abundance of bacterial phyla across %FGV terciles. Global and pairwise p-values are presented from Kruskal-Wallis and Wilcoxon tests, respectively. Ns = non-significant at an alpha level of 0.05 (p>0.05); *: p<= 0.05; **: p<= 0.01
