# Supplementary figures and images for: The Association Between Breast Density and Gut Microbiota Composition at 2 Years Post-Menarche: A Cross-Sectional Study of Adolescents in Santiago, Chile
Source: Front Cell Infect Microbiol. 2021 Dec 17;11:794610. doi: 10.3389/fcimb.2021.794610 (PMC8718921; doi:10.3389/fcimb.2021.794610)

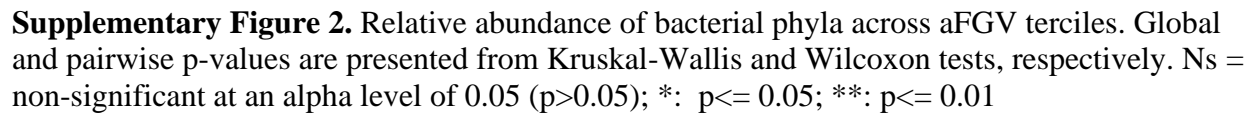

Supplement: Supplementary file 2 [file Image_2.pdf]
